# The V5-Epitope Tag for Cell Engineering and Its Use in Immunohistochemistry and Quantitative Flow Cytometry

**DOI:** 10.3390/biology14070890

**Published:** 2025-07-20

**Authors:** Katja Fritschle, Marion Mielke, Olga J. Seelbach, Ulrike Mühlthaler, Milica Živanić, Tarik Bozoglu, Sarah Dötsch, Linda Warmuth, Dirk H. Busch, Arne Skerra, Christian Kupatt, Wolfgang A. Weber, Richard E. Randall, Katja Steiger, Volker Morath

**Affiliations:** 1 Department of Nuclear Medicine, TUM University Hospital, School of Medicine and Health, Technical University of Munich, 81675 Munich, Germany; 2 Comparative Experimental Pathology (CEP), School of Medicine and Health, Technical University of Munich, 81675 Munich, Germany; 3 Deutsches Zentrum für Herz-Kreislaufforschung (DZHK), 80636 Munich, Germany; 4 Medizinische Klinik I, School of Medicine and Health, Technical University of Munich, 81675 Munich, Germany; 5 Institute for Medical Microbiology, Immunology and Hygiene, School of Medicine and Health, Technical University of Munich, 81675 Munich, Germany; 6 Lehrstuhl für Biologische Chemie, School of Life Sciences, Technical University of Munich, 85354 Freising, Germany; 7 Bavarian Cancer Research Center, Partner Site Munich, 91052 Erlangen, Germany; 8 Centre for Biomolecular Sciences, School of Biology, University of St. Andrews, St. Andrews KY16 9ST, UK

**Keywords:** DTPA-R, immunohistochemistry, immuno-engineering, synthetic biology, V5 tag

## Abstract

Synthetic biology has fundamentally advanced cell engineering and helped to develop effective therapeutics such as chimeric antigen receptor (CAR)-T cells. For these applications, the detection, localization, and quantification of heterologous fusion proteins assembled from interchangeable building blocks is of high importance. The V5 tag, a 14-residue epitope tag, offers promising characteristics for these applications but has only rarely been used in this context. Thus, we have systematically evaluated the murine anti-V5 tag antibody mu_SV5-Pk1 as well as its humanized version, hu_SV5-Pk1, to analyze cells expressing V5-tagged receptors in samples from various in vitro and in vivo experiments. We found that the V5 tag signal on cells is affected by certain fixation and detachment reagents. Immunohistochemistry (IHC) on formalin-fixed paraffin-embedded (FFPE) mouse tissue samples was performed to sensitively detect cells in tissue. We improved IHC by applying the hu_SV5-Pk1 monoclonal antibody (mAb) to avoid cross-reactivity within and unspecific background signals arising on fixed mouse tissue. Conversely, the absence of unspecific binding by the mu_SV5-Pk1 mAb was evaluated on 46 human normal or cancer tissues. Our findings present a robust toolbox for utilizing the V5 tag and cognate antibodies in synthetic biology applications.

## Simple Summary

Developing new therapies based on modified cells requires the detection and analysis of these modified cells by reliable methods. This study investigates and improves the use of a small marker, the so-called V5 tag, which can be incorporated into any protein of interest. Two types of antibodies that can bind the V5 tag—one from mice and one adapted to the human antibody structure—were investigated in this study. It was examined how well these antibodies worked on different samples, including cells and tissue samples from mice. It was found that certain chemicals used in preparing samples could compromise the ability to detect the tag. Also, it was shown how to reduce background signals that can complicate the analysis, especially in mouse tissues. This work provides reliable methods for using the V5 tag in biological research, which can support the development of future medical treatments.

## 1. Introduction

Protein tags, initially used to study the role of proteins in cells [1], have been extensively used for various purposes across life science research [2,3,4]. Usually, they are short peptides with specific sequences that can be genetically encoded within a fusion protein, thus providing binding sites (epitopes) for antibodies or alternative binding proteins [5]. Multiple criteria need to be considered when choosing an epitope tag for a specific application. These include (1) molecular size; (2) hydrophilicity, total charge and charge distribution (which may affect membrane integration, see Table S1 for commonly used tags); (3) pre-existing or potential immunogenicity in species in case of in vivo application; (4) susceptibility to proteolytic cleavage; (5) absence of cross-reactivity with other epitopes found in the proteome; and, importantly, (6) the availability of high-affinity antibodies and staining protocols for the detection of the epitope tag. Moreover, the use of multiple established epitope tags permits the orthogonal multiplexed detection of fusion proteins.

Such tags can be used in synthetic biology applications that aim to create new biological functions, for example, engineered cell therapies. One of these are the so-called chimeric antigen receptors (CARs) that enable the re-direction of genetically modified T cells to tumor cells and their activation independently of the T cell receptor (TCR) [6]. CAR-T cell therapies have shown dramatic efficacy in some leukaemias, and there is enormous interest in applying these therapies on a broader spectrum of diseases. Beyond cell therapies, tags can be useful in the development and characterization of gene therapies. In this setting, a gene is therapeutically delivered using a vehicle, for example, adeno-associated virus (AAV), to treat various diseases. Here, the tag could help evaluate the efficacy of gene delivery. Research in these fields can be greatly facilitated by epitope tag platforms that can ideally be used throughout the drug development process.

In principle, the V5 tag (NH_2_-GKPIPNPLLGLDST-COOH) offers several promising characteristics that make it attractive for studying cell and gene therapy throughout the drug development process but has not yet been broadly used in this context. Its sequence comprises residues 95 to 108 of the P-subunit of simian virus 5 (SV5) RNA polymerase [7,8,9]. Different high-affinity antibodies directed against the V5 tag as epitope are available, including the monoclonal mouse IgG2a antibody SV5-Pk1 (mu_SV5-Pk1), which exhibits an affinity of ~20 pM and recognizes the core motif PNPLL [9]. In addition, a nanobody with an intermediate affinity of around 29 nM, which binds over the full length of the V5 tag (Gly^1^ to Thr^14^), was developed for studying protein-protein interactions in cells [10].

The V5-epitope tag system has been used for various applications outside CAR-T cell research, including the purification of recombinant proteins [11]; studies of ion channels [12] and topological investigations of membrane proteins [13]. Furthermore, it was demonstrated that cells displaying the V5 tag can be identified on tissue sections by chromogenic immunohistochemistry (IHC) as well as immunofluorescence (IF) [14,15]. A beneficial property of the V5 tag relates to its low hydrophilicity; therefore, it is not expected to affect the translocation of proteins into the biological membrane, which is critical for studies on membrane-bound CARs [16,17,18].

**Figure 1:**
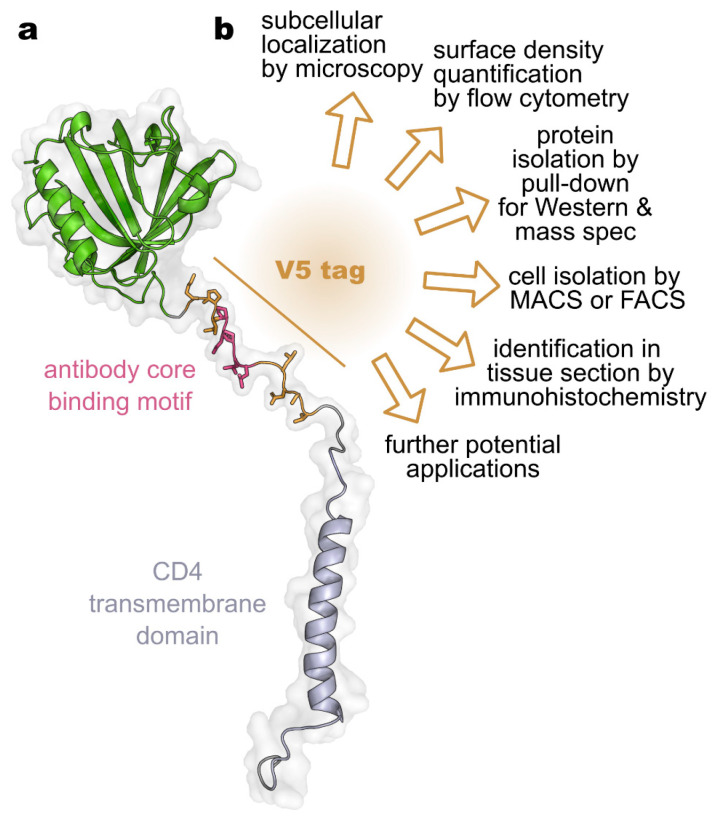
(**a**) Schematic representation of the V5 tag in the synthetic membrane protein DTPA-R developed for PET imaging [19]. The reporter protein consists of an Anticalin (green) binding CHX-A″-DTPA•metal chelates, an extracellular linker containing the V5-epitope tag (ochre, core motif in purple), and the CD4 transmembrane domain. (**b**) Potential use cases for the V5 tag.

Here, we describe the characterization and optimization of the V5-epitope tag system for the development of a reporter gene encoding a fusion protein that allows the quantitative assessment of CAR-T cell distribution and AAV-mediated gene transfer in live animals via positron emission tomography (PET) imaging [19]. For these applications, we introduced a membrane-anchored CHX-A″-DTPA•metal binding protein (DTPA-R, Figure 1a) as PET reporter gene, which binds the radioligand [^18^F]F-DTPA, and used the V5 tag to measure the expression of the reporter gene in vitro and to verify PET results in mice using some of the available methods (Figure 1b) [19]. Now, we report the characterization and optimization of two anti-V5 tag antibodies for flow cytometric and histochemical detection, which should facilitate their application in future synthetic biology studies.

## 2. Materials and Methods

### 2.1. Cell Culture

Eucaryotic cells were cultured at 37 °C in a humidified 5% CO_2_ atmosphere and were regularly tested by PCR for potential mycoplasma contamination. The Jurkat T cell line was obtained from Prof. Bernhard Küster, TU Munich (American Type Culture Collection (ATCC), Manassas, VA, USA; TIB-152), and the Raji cell line was obtained from Prof. Stanley Riddell, Fred Hutchinson Cancer Center Seattle (ATCC: CCL-86). Peripheral blood mononuclear cells (PBMCs) were isolated from blood from a healthy donor (German Red Cross Blood Donor Service, Munich, Germany) via density gradient centrifugation as described before [19]. Generation and culture of Jurkat^DTPA-R^ and CAR-T^DTPA-R^ cells stably expressing the DTPA-R reporter protein has been described before [19]. All Jurkat cell lines and Raji cells were cultured in Roswell Park Memorial Institute (RPMI) 1640 medium with GlutaMAX supplement, 10% (*v*/*v*) fetal bovine serum (FBS) and 1% (*v*/*v*) penicillin/streptomycin (pen/strep) stock solution (10,000 U/mL/10 mg/mL; all from Gibco, Thermo Fisher Scientific, Waltham, MA, USA).

### 2.2. Cell Fixation

Jurkat^DTPA-R^ and Jurkat wildtype control cells were counted and stained with zombie violet live-dead stain (1:1000 dilution, BioLegend, San Diego, CA, USA) for 20 min at room temperature and subsequently washed once with FACS buffer (5% FBS in Dulbecco’s phosphate-buffered saline (DPBS), Gibco) and once with DPBS. 3 × 10^6^ cells were pelleted and carefully resuspended in 1 mL fixative, being either 4% paraformaldehyde (PFA, Merck, Darmstadt, Germany; dissolved in DPBS at 60 °C under addition of NaOH till dissolved, pH adjusted to 6.9 with HCl), neutral-buffered 4% formaldehyde solution (Otto Fischar, Saarbrücken, Germany), PAXgene Tissue FIX (PreAnalytiX, Homberchtikon, Switzerland) or −20 °C cold 80% EtOH (Merck; in DPBS). Samples were incubated at room temperature (PFA, formaldehyde, PAXgene) or −20 °C (EtOH) for 30 min or 24 h. After incubation, samples fixed with PAXgene were centrifuged at 300× *g* for 3 min and resuspended in PAXgene STABILIZER for 10 min before further processing. After incubation, all samples were washed three times with 1 mL DPBS and once with FACS buffer before processing as described in “quantitative flow cytometry”.

### 2.3. Cell Detachment

Collagenase for cell detachment was freshly prepared as follows: Collagenase II (Worthington Biochemical Corporation, Lakewood, NJ, USA) was dissolved in Hank’s Balanced Salt Solution (HBSS; Gibco), and collagenase IV (Merck) was dissolved in Dulbecco’s Modified Eagle Medium (DMEM; Gibco), both with a concentration of 1.5 mg/mL. Papain digestion was performed as previously described [20]. In short, papain (40 U/mL; Worthington Biochemical Corporation) with 2 mM L-cysteine (Sigma-Aldrich, St. Louis, MO, USA) in DPBS was activated at 37 °C for 10 min. After incubation, the solution was diluted 1:2 with DPBS to obtain the 1× solution. Trypsin-EDTA (0.25%, Gibco) and Accutase (Gibco) were ready-to-use solutions.

Jurkat^DTPA-R^ cells were counted and stained with zombie violet live-dead stain (1:1000 dilution, BioLegend) for 20 min at room temperature and subsequently washed with FACS buffer. 3 × 10^6^ cells were used for each treatment. Cells for trypsin and Accutase detachment were washed with DPBS; for papain digestion with 2 mM EDTA in DPBS; for collagenase II with HBSS; and for collagenase IV with DMEM. After washing, cells were resuspended in 600 µL of the respective cell detachment reagent. A sample of these cell suspensions was taken at each time point, and the cell detachment was stopped with the respective stop solution. The stop solution for trypsin, Accutase, collagenase II, and IV was FACS buffer with complete Ultra protease inhibitor with EDTA (1 tablet per 10 mL; Roche, Basel, Switzerland). For papain, the digestion was stopped in 200 µL stop solution (1 mg/mL trypsin inhibitor, Sigma Aldrich and 16 µL DNase I (5 mg/mL stock, Sigma-Aldrich) in DPBS). Samples were stained and analyzed as described in “quantitative flow cytometry” or subjected to a radioligand binding assay.

### 2.4. Antibody Labeling with Fluorochrome

Murine SV5-Pk1 antibody (Bio-Rad, Hercules, CA, USA) was conjugated with a fluorescent dye; therefore, 600 µg (4 nmol) antibody was dialyzed against carbonate buffer (100 mM NaCO_3_, pH 8.6) and mixed with a 20-fold excess of AlexaFluor 488 (AF488, Lumiprobe, Hannover, Germany; in DMSO) at room temperature overnight. The reaction mixture was loaded on a PD-10 column (Cytiva, Marlborough, MA, USA) equilibrated with SA-buffer (100 mM Tris/HCl pH 8.0, 150 mM NaCl, 1 mM EDTA), and the eluate was collected in 500 µL fractions. The absorbance of the fractions was measured using a NP80 NanoPhotometer (Implen, Munich, Germany), and the three fractions with the highest antibody concentration were pooled. Concentration and DOL of the pooled fractions were measured in quintuplicates, and the mean values were used for further calculations.

### 2.5. Quantitative Flow Cytometry

The absolute number of receptors per cell was determined as described previously [19]. In short, cells were stained with anti-V5 tag antibody clone SV5-Pk1 (Bio-Rad) conjugated to AF488 (2.9 µg/mL) on ice for 1 h. After three washing steps with FACS buffer, cells were resuspended in 100 µL FACS buffer and analyzed on a LSR-Fortessa flow cytometer (Becton Dickinson, Franklin Lakes, NJ, USA) using 405 and 488 nm excitation lasers and bandpass filters for BV421 (450/40 nm) and FITC (530/30 nm). The Quantum MESF kit Alexa Fluor 488 (Bangs Laboratories, Fishers, IN, USA) beads were analyzed in the flow cytometer on the same day in FACS buffer. Results were analyzed using FlowJo software (ver. 10.8.1; Becton Dickinson). Median FITC fluorescence was normalized to untreated Jurkat^DTPA-R^ cells.

### 2.6. Radioligand Binding Assay

The radioligand [^18^F]F-DTPA was prepared as previously described [19] and will be described in detail elsewhere. In short, [^18^F]F^−^ was eluted with 700 µL 75 mM tetrabutylammonium (TBA) hydroxide solution from an anion exchange Sep-Pak QMA carbonate Plus Light cartridge (Waters, Milford, MA, USA) on a Modular-Lab Standard synthesis module (Eckert & Ziegler, Berlin, Germany). After drying at 95 °C for 5 min and two azeotropic drying steps using anhydrous acetonitrile (0.001% H_2_O max.; Merck Millipore, Burlington, MA, USA) for 5 min, the precursor TMA-Nic-D-Glu_2_-PEG_4_-CHX-A″-DTPA (0.5 mg in 500 µL anhydrous dimethyl sulfoxide (DMSO, 0.005% H_2_O max.; VWR, Radnor, PA, USA)) was added. After 10 min labeling at 95 °C, the mixture was diluted with deionized H_2_O, and the product was separated on a 250 × 4.6 mm C18 reversed-phase HPLC column (ReproSil C18 Aq, 5 µm particle size; Dr. A. Maisch, Ammerbuch, Germany) using an isocratic elution with 25% MeCN with 0.1% TFA as the mobile phase. The product was further purified using a Sep-Pak C18 classic cartridge (Waters) and eluted with 1 mL EtOH. 200 µL of a 0.15 M NH_4_OAc buffer pH 5.5 with 20 mM terbium^III^ chloride hexahydrate (AlfaAesar, Haverhill, MA, USA) and 200 µL deionized H_2_O was added, and complexation was completed at 55 °C for 15–30 min until the ethanol evaporated. Thereafter, 1 mL DPBS was added, and the precipitated free terbium was pelleted by centrifugation. The supernatant containing the [^18^F]F-Nic-D-Glu_2_-PEG_4_-CHX-A″-DTPA•Tb radioligand ([^18^F]F-DTPA) was used for further experiments.

Jurkat cells were prepared as described under “cell detachment”, and 1 × 10^6^ cells (without antibody stain) were pelleted and resuspended in PBS_mod_ (PBS with 10 g/L CaCl_2_, 10 g/L MgCal_2_•6 H_2_O, 2% BSA) containing 0.5 MBq/mL [^18^F]F-DTPA. Cells were incubated on ice for 1 h and subsequently washed with PBS_mod_ buffer four times. After washing, the cells were lysed with 1 M NaOH, and radioactivity was quantified using a Wizard^2^ automated gamma counter (PerkinElmer, Waltham, MA, USA). Data were normalized to untreated Jurkat^DTPA-R^ cells at 100%.

### 2.7. Animal Experiments

Animal experiments were conducted as previously described [19]. Mice were housed in a specific-pathogen-free (SPF) environment in Sealsafe Next Greenline individually ventilated cages (IVC; Techniplast, Buguggiate, Italy) under a 12 h day–night cycle with access to ad libitum chow and water. Mouse strains C57BL/6 (C57BL/6NCrl, strain code 027), CD1-nude (Crl:CD1-*Foxn1^nu^*, strain code 086), and NSG (NOD.Cg-Prkdc^SCID^Il2rg^tm1Wjl^/SzJ, strain code 614) were purchased from Charles River Laboratories (Sulzfeld, Germany). All animals were allowed a one-week acclimatization period. Animal experiments were conducted in accordance with institutional guidelines and animal welfare regulations in Germany (permission from the District Government of Upper Bavaria approval ROB-55.2-2532.Vet_02-21-41 and Vet_216-15). Humane endpoints were defined that included, among other criteria, the loss of 10% body mass compared to the previous week. The results are traceable by unique institutional animal numbers (#xxx). Female animals were used to decrease biological variation. Researchers were not blinded during animal studies or data analysis.

AAV9 viral vectors encoding an expression cassette for DTPA-R (AAV9^DTPA-R^; produced as described in [19]) were intravenously injected via the tail vein into CD1-nude or C57BL/6 mice with lowest (1 × 10^11^ viral genomes (vg)/mouse), low (5 × 10^11^ vg/mouse), high (1 × 10^12^ vg/mouse), or highest (2.5 × 10^12^ vg/mouse) dose. [^18^F]F-DTPA PET/MR imaging was performed 11 days post AAV injection.

CAR-T cell distribution and proliferation was followed in NSG mice engrafted with 5 × 10^5^ Raji-fLuc-GFP^+^ cells via tail vein injection as described in [19]. After seven days, 2 × 10^6^ sorted CAR-T cells expressing the reporter protein DTPA-R were intravenously injected. [^18^F]F-DTPA PET/MR imaging was performed on days 1, 4, 8, and 14 after CAR-T cell administration. The animal was sacrificed on day 15, and the tissue was prepared for histological evaluation or flow cytometry.

### 2.8. PET/MR Imaging

Imaging was conducted as described in [19]. In short, the animals received intravenously 10 to 12 MBq of [^18^F]F-DTPA and were kept awake until the PET imaging started at t = 90 min for 20 min. PET/MR acquisition was performed with a nanoScan PET/MR system with 3T field strength and two PET rings (Mediso Medical Imaging Solutions, Budapest, Hungary) operated with Nucline NanoScan software (ver. 3.04.025.0000; Mediso). T1-weighted MRI images were recorded using a 2D FSE sequence.

### 2.9. Preparation of Cell Pellets of Jurkat^DTPA-R^ and Jurkat Wildtype Cells as Positive and Negative Control

Per cell line (Jurkat^DTPA-R^ or Jurkat wildtype), one 175 cm^2^ culture flask was harvested, washed with PBS, and cells were pelleted and fixed in 4% formalin solution for 15 min. Formalin supernatant was discarded, and cells were washed twice with PBS. Cells were resuspended in 200 µL of 1% (*w*/*v*) agarose solution in PBS, and cells were transferred into the lid of a 1.5 mL microcentrifuge tube. The generated cell pellet was placed in a histological embedding cassette, dehydrated according to standard protocols using an automated system (ASP300S; Leica Biosystems, Nussloch, Germany), and embedded in paraffin. 2 µm sections of a positive and a negative control were placed on one glass slide to serve as IHC controls.

### 2.10. Immunohistochemistry

Tissue samples from animal experiments were fixed in neutral-buffered 4% formaldehyde solution for 48 h at room temperature and subsequently transferred into PBS and stored at 4 °C. The spine was decalcified in Osteosoft (Merck Millipore) for 27 days. The tissues were dehydrated using an automated system (ASP300S; Leica Biosystems) and embedded in paraffin. Serial 2 µm sections were cut using a rotary microtome (HM355S; Thermo Fisher Scientific) and deparaffinized using deparaffinization solution (Leica Biosystems). The tissue microarrays 23 Core Cancer Human Tissue Microarray (BSB 0231), 23 core Human Normal Tissue Microarray (BSB 0298), and 31 Core Human Cancer Cell Line Microarray (BSB 0244) (all from Bio SB, Santa Barbara, CA, USA) were also deparaffinized using deparaffinization solution (Leica Biosystems). Immunohistochemistry from formalin-fixed paraffin-embedded (FFPE) samples (IHC(P)) was performed using a Bond RXm system (Leica Biosystems) starting with blocking in 3% hydrogen peroxide solution and, if indicated, with 3% normal goat serum (Abcam, Waltham, MA, USA) as protein blocking reagent. The primary antibodies used were murine anti-V5 tag antibody mu_SV5-Pk1 (clone SV5-Pk1; 1:500; Bio-Rad), polyclonal rabbit anti-V5 tag antibody (600-401-378; Thermo Fisher Scientific), rabbit polyclonal anti-V5 tag antibody (orb345390; biorbyt, Durham, NC, USA) or humanized anti-V5 tag antibody hu_SV5-Pk1 (clone SV5-Pk1; 1:800; obtained from Prof. Randall, St. Andrews University, UK) and rabbit anti-human CD19 antibody (clone D4V4B; 1:600; Cell Signaling Technology, Danvers, MA, USA). In brief, after antigen retrieval with epitope retrieval solution 1 (corresponding to citrate buffer, pH 6) for 30 min, the primary antibody was incubated at given dilutions for 15 min. The slides were subsequently incubated with the polymer refine and/or refine red detection kit without post-primary reagent (Leica Biosystems; for anti-CD19 antibody and polyclonal rabbit anti-V5 tag antibodies) or with an intermediate rabbit anti-mouse (<10 µg/mL, Leica Biosystems; for mu_SV5-Pk1) or rabbit anti-human IgG (polyclonal; 1:200; Jackson ImmunoResearch Laboratories, Ely, UK; for hu_SV5-Pk1) bridging antibody for 8 min at room temperature. The secondary anti-rabbit Poly-HRP-IgG (<25 µg/mL, Leica Biosystems) was detected with DAB (3,3′-diaminodbenzidine; Leica Biosystems) as the colorimetric substrate or Fast Red chromogen included in the refine red detection kit (Leica Biosystems). All IHC slides were counterstained using hematoxylin (Leica Biosystems). Slides were scanned using an Aperio AT2 digital pathology slide scanner, and representative image regions were prepared using Aperio ImageScope (ver. 12.4) software (both Leica Biosystems). Signals classified as unspecific by an experienced pathologist were not included. The positive cell fraction was analyzed using QuPath (ver. 0.3.2) software [21].

### 2.11. Data Analysis and Figure Preparation

PET data were analyzed using Inveon Research Workplace (ver. 4.2; Siemens Medical Solutions, Knoxville, TN, USA). IHC staining was visualized using Aperio ImageScope (ver. 12.4, Leica Biosystems) and analyzed using QuPath [21] (ver. 0.3.2). Figures were assembled using Inkscape (ver. 1.2.1; www.Inkscape.org). Image processing was performed with GIMP (ver. 2.10.30; www.gimp.org). Data visualization was performed using GraphPad Prism software (ver. 9.3.1; GraphPad, San Diego, CA, USA). Protein structures were visualized using PyMol (ver. 2.5.2; Schrödinger, New York, NY, USA).

## 3. Results

### 3.1. Quantification and Analysis of Fusion Protein Expression

**Figure 2:**
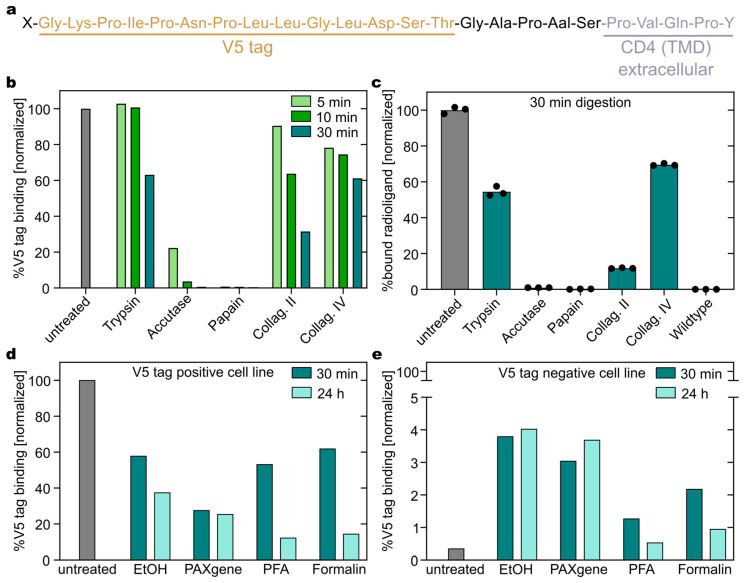
Influence of cell detachment and fixation on detectable V5 tag signal. (**a**) Extracellular linker sequence of DTPA-R including the V5 tag (ochre) between the Anticalin binding protein (X) and the CD4-transmembrane domain (Y). (**b**,**c**) Jurkat^DTPA-R^ cells were treated with different cell detachment reagents. (**b**) The V5 tag signal (stained with mu_SV5-Pk1-AF488) was measured by flow cytometry after different incubation times (median fluorescence intensity (MFI)). (**c**) [^18^F]F-DTPA radioligand binding of Jurkat^DTPA-R^ cells treated with different cell detachment reagents for 30 min was analyzed to detect potential cleavage in the binding protein. Untreated Jurkat^DTPA-R^ cells (100% binding) and Jurkat wildtype cells served as references (mean of triplicates in one experiment in panel c). (**d**) Jurkat^DTPA-R^ and (**e**) V5 tag negative Jurkat wildtype cells were fixed with different reagents, and the V5 tag signal (stained with mu_SV5-Pk1-AF488) was analyzed by flow cytometry.

When quantitatively assessing the expression of a fusion protein that carries an epitope tag, it is crucial to consider the details of technical preparation, as this may affect the detected signals. Many procedures require dissociation of cells from adherent culture or from tissue samples and/or the fixation of cells or tissues. A multitude of reagents are available for both techniques. To assess the effect of commonly used dissociation and fixation reagents on V5 tag signal, we employed the previously described DTPA-R reporter protein [19] as a model membrane protein. In this reporter protein, the V5 tag is located extracellularly in close proximity to the cell membrane (9 amino acids), forming part of the linker between the transmembrane helix and the binding protein that recognizes CHX-A″-DTPA•metal complexes (Figure 2a).

The influence of five commonly employed protease-based cell detachment reagents on the immunochemical detection of the V5 tag and the integrity of the entire DTPA-R reporter on Jurkat^DTPA-R^ cells was investigated by flow cytometry using the directly fluorescently labelled antibody mu_SV5-Pk1-AF488 and a radioligand binding assay (Figure 2b,c). In addition, three different incubation times were evaluated by flow cytometry to study the rate of proteolytic cleavage of the tag. Accutase and papain cleaved the V5 tag quantitatively already at short incubation times ≤ 5 min. In contrast, the V5 tag signal remained detectable at roughly 50% signal strength even after 30 min incubation when using collagenases belonging to type II (also referred to as matrix metalloproteinase (MMP) 8 [22]) or to type IV (also referred to as MMP2 [23]) (Figure 2b). Trypsin treatment resulted in the best preservation of the V5 tag among the tested proteases (Figure 2b).

The functional integrity of the membrane-associated DTPA-R reporter protein was assayed after 30 min protease digestion using a radioligand binding assay, thus investigating potential cleavage at the N-terminal side of the V5 tag. Radioligand binding activity of the DTPA-R was proportional to the detected V5 tag signal as described above, with trypsin and collagenase VI treatments leading to the best preservation (Figure 2c). Taken together, trypsin was identified as a reagent that allows the convenient detachment of cells without significant loss of signal in the immunochemical detection of the V5 tag, at least for incubation times up to 10 min, which is mostly sufficient for cell detachment [24].

Next, we assessed the influence of fixatives on the detectable signal for the V5 tag (Figure 2d). The fixatives explored were as follows: (i) cold (−20 °C) 80% (*v*/*v*) ethanol; (ii) PAXgene tissue fix; (iii) freshly prepared 4% (*w*/*v*) formaldehyde from paraformaldehyde (PFA); and (iv) methanol-stabilized 4% (*w*/*v*) formalin. The V5 signal was detected after 30 min or 24 h incubation with the fixative. After 30 min fixation, all fixatives led to a decreased signal (Figure 2d); however, the effect was most pronounced for PAXgene, with a remaining signal of 38%. Interestingly, the impact of the PAXgene fixation did not substantially change upon continued fixation, whereas all other fixatives led to an increased signal loss after 24 h, up to 88% (Figure 2d). Furthermore, incubation of cells with fixatives slightly increased the unspecific binding of the anti-V5 tag antibody mu_SV5-Pk1, thereby increasing the fluorescence intensity signal relative to the untreated Jurkat^DTPA-R^ cells signal from 0.4% to up to 3.8% (Figure 2e).

**Figure 3:**
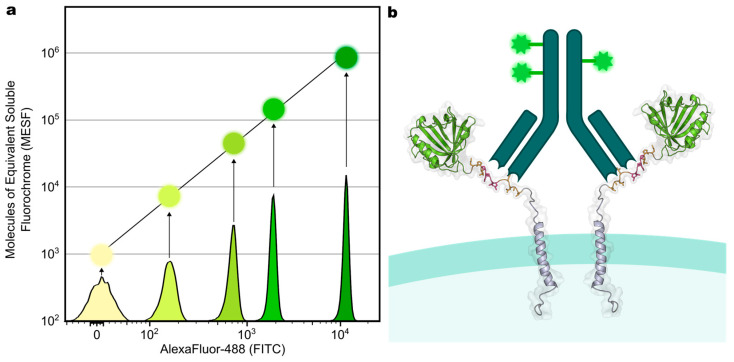
(**a**) Quantification of receptor numbers by interpolation from flow cytometry measurements. For each bead population (labelled with different amounts of AF488), median FITC values are obtained by flow cytometry and correlated with the molecules of equivalent soluble fluorochrome (MESF) values provided by the manufacturer. The resulting regression line is used to calculate the MESF values for cell populations of interest. (**b**) For calculating the number of receptors per cell, the obtained MESF value of the cell population and the DOL for the antibody are used. It is assumed that one antibody binds two receptors simultaneously.

Following the characterization of the cell preparation conditions, we applied the V5 tag to quantify DTPA-R densities (number of receptors per cell) by flow cytometry. Commercial kits based on beads with known quantities of fluorophore (e.g., Bangs Laboratories Quantum MESF kit) can be used to convert fluorescence intensity signals measured upon binding of an antibody with known degree of labelling (DOL) to an absolute number (Figure 3a). However, the assumption of one (bivalent) antibody binding two receptors (Figure 3b) might lead to an overestimation of receptor numbers, especially in the case of low expression levels, which promotes monovalent complex formation. This could be circumvented by using monovalent binders such as the anti-V5 tag nanobody [10], a single-chain variable fragment (scFv) or proteolytic Fab fragment of SV5-Pk1 antibodies. Nevertheless, this method allowed us to quantify the receptor number on CAR-T cells expressing the DTPA-R reporter gene. In particular, ~86,000 copies of DTPA-R were detected using the V5 tag (antibody mu_SV5-Pk1-AF488: DOL 3.8), while ~170,000 copies of CD3 (anti-CD3-AF488: DOL 3.9), ~150,000 copies of CD4 (anti-CD4-AF488: DOL 5.8), and ~17,200 copies of CXCR3 (anti-CXCR3-AF488: DOL 3.9) were detected on the cell surface using commercial receptor-specific antibodies [19].

### 3.2. Establishing Immunohistochemistry for the V5 Tag in Mice

Besides the quantitative comparison of V5-tagged proteins at the single-cell level using flow cytometry, IHC on tissue sections is another important method to detect engineered cells within tissues. Indeed, IHC using the V5 tag can unequivocally determine cell identity and provide spatial information about the location of cells and their interaction with cells in proximity, quantify relative cell numbers and provide macroscopic information about tissue sections.

**Figure 4:**
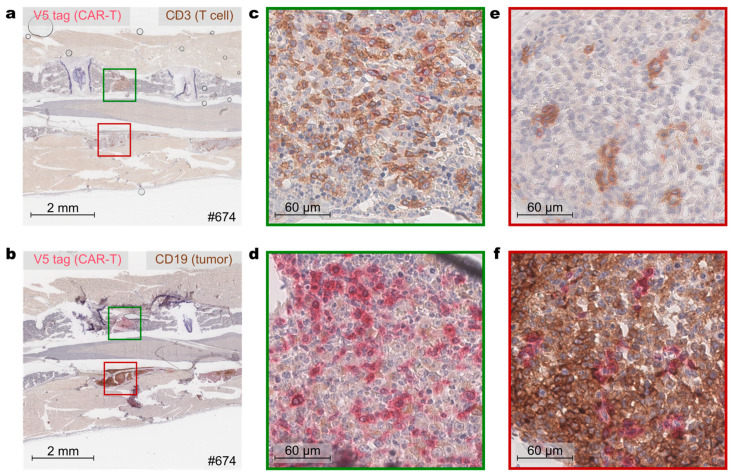
Exemplary IHC using the mu_SV5-Pk1 antibody on spine samples from Raji lymphoma-bearing NSG mice treated with CAR-T^DTPA-R^ cells. (**a**,**b**) IHC co-staining of consecutive spine sections stained for CAR-T^DTPA-R^ cells using the anti-V5 tag (Fast Red, pink) together with (**a**) an anti-CD3 staining or (**b**) an anti-CD19 (specific for tumor cells) staining with DAB (brown). (**c**–**f**) Higher magnifications of tumor lesions with (**e**,**f**) low (red outline) or (**c**,**d**) high (green outline) infiltration of CAR-T^DTPA-R^ cells in the vertebra. Co-staining for the lymphoma-specific marker CD19 showed (**d**) low tumor cell number with high CAR-T cell number, indicating clearance of the lesion as opposed to a (**f**) high number of tumor cells with only a few CAR-T cells.

An example of such an IHC analysis is depicted for a CAR-T cell study, where immunocompromised NSG mice, a mouse strain based on NOD/SCID with complete null mutation of the interleukin 2 receptor (IL2r) γ chain [25,26], bearing a CD19-positive lymphoma were treated with CAR-T cells co-expressing the reporter protein DTPA-R. IHC of the spine of one of these animals clearly showed V5 tag positive CAR-T cells with distinct membrane staining and low signal background in two separate regions (Figure 4). V5-IHC was complemented with either anti-CD3 staining (Figure 4a,c,e) to confirm the identity of CAR-T cells using a proven T cell marker, or with anti-CD19 staining (Figure 4b,d,f) to investigate the co-localization with lymphoma tumor cells. The upper lesion (Figure 4c,d) showed a high amount of V5 tag positive T cells and only a few tumor cells, indicating successful clearing of the lesion by the therapeutic CAR-T cells. On the other hand, the lower lesion (Figure 4e,f) revealed a high density of CD19-positive tumor cells with only a few CAR-T cells infiltrated.

### 3.3. Optimization of V5 Tag Immunohistochemistry

**Figure 5:**
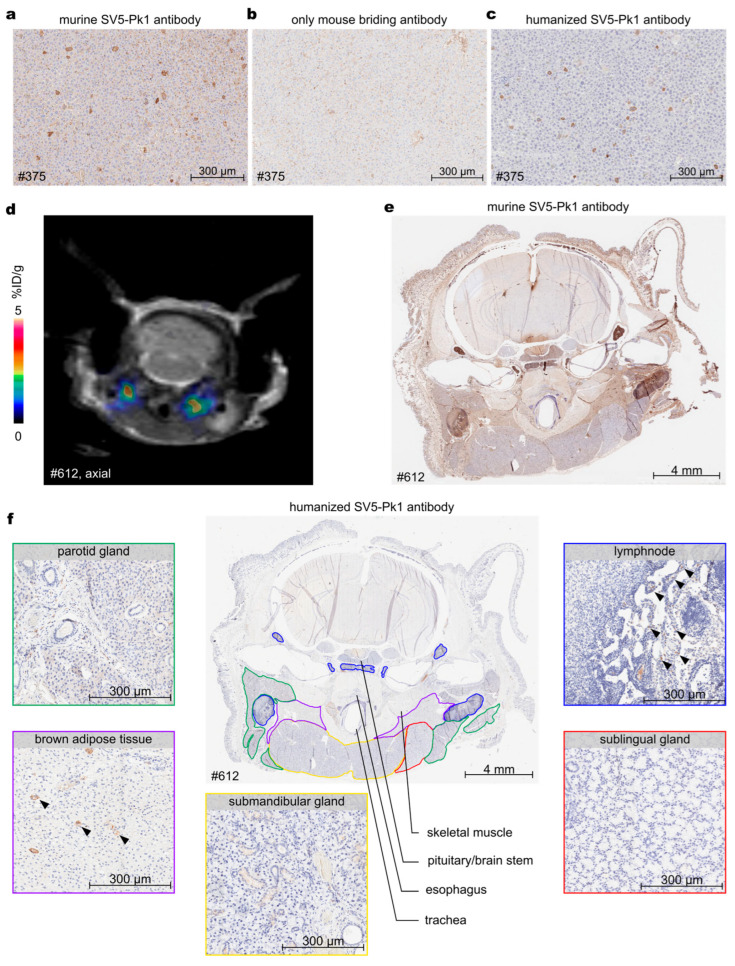
IHC of tissues from CD1-nude mice transduced with AAV9^DTPA-R^ viral vectors. (**a**) IHC using mu_SV5-Pk1 antibody (1:500) and rabbit anti-mouse bridging antibody (post-primary reagent BONDx system) showed extracellular background staining. (**b**) IHC using only rabbit anti-mouse bridging antibody detecting background signals. (**c**) IHC using the humanized anti-V5 tag antibody hu_SV5-Pk1 (1:800) and a rabbit anti-human bridging antibody led to reduced background signal. (**d**) Axial PET/MR image of mouse treatment with AAV9^DTPA-R^ viral vectors (1 × 10^11^ vg/mouse) acquired 90 min post intravenous radioligand ([^18^F]F-DTPA) injection. The section shows a cross-sectional plane of the head of the mouse with PET signal in the thoric region of the neck. (**e**) IHC of mouse head (sacrificed 34 days after AAV9^DTPA-R^ injection) section to verify PET results stained with mu_SV5-Pk1 showing high background signal. (**f**) Consecutive section was stained with hu_SV5-Pk1, yielding a drastically reduced background and specific V5 tag positive signal in the brown adipose tissue and positive staining of single cells in the lymph nodes (black arrowheads).

NSG mice are highly immunocompromised and completely lack humoral immunity. Consequently, there are no host antibodies that could interfere with V5 tag detection using a murine monoclonal antibody in IHC [25]. However, the less immunocompromised CD-1 nude mice, another commonly used mouse strain for biomedical research, feature B cells, thus challenging this kind of IHC. We used CD-1 nude mice to follow gene transfer by AAV9 viral vectors using the DTPA-R reporter gene. In these mice, we observed predominantly extracellular staining of liver tissue, indicating non-specific antibody binding, which was verified by an IHC control without the anti-V5 tag primary antibody (Figure 5a,b). In contrast, a similar extracellular staining was not visible in the liver or kidney tissue of NSG mice (Figure S1). Our first attempt to optimize the anti-V5 IHC made use of 3% normal goat serum as a protein blocking reagent to reduce the unspecific signal due to the endogenous Fc-domains, but was not successful.

Thus, we decided to try different primary anti-V5 tag antibodies from rabbits to reduce background staining, which was most likely due to the anti-mouse bridging antibody detecting murine IgG and IgM [27]. Using formalin-fixed and paraffin-embedded pellets of Jurkat^DTPA-R^ cells expressing the V5 tag and their corresponding Jurkat wildtype control cells, these antibodies were tested for applicability and target specificity. However, two polyclonal rabbit anti-V5 tag antibodies (from Thermo Fisher Scientific and biorbyt, respectively) offered for IHC applications did not work in our hands on FFPE fixed cell pellets.

Finally, we tested the recently described humanized monoclonal IgG1 antibody clone SV5-Pk1 (hu_SV5-Pk1) [28]. This reagent allowed the use of a rabbit anti-human bridging antibody, which does not cross-react with murine antibodies. This procedure led to the successful identification of V5 tag positive cells in the FFPE cell pellet and also of V5 tag positive cells in murine liver tissue (due to AAV9 transduction) without the aforementioned limitations (Figure 5c).

Another example where a low background signal is important became evident when searching within the throat region of a CD-1 nude mouse for tissue that had been transduced by AAV9 viral vectors delivering the DTPA-R gene, thus causing pronounced radioligand enrichment in a PET image (Figure 5d). These clear but localized PET signals could not be assigned to specific anatomic structures using PET/MR and consequently necessitated histological investigation. However, the initially applied mu_SV5-Pk1 antibody produced a high background signal in various tissues (Figure 5e), making reliable interpretation impossible, even for an experienced pathologist. In marked contrast, the new hu_SV5-Pk1 antibody in combination with the rabbit anti-human bridging antibody did not cause such background signals (Figure 5f). In the region of interest, different glands and tissues were annotated, and V5 tag staining was identified in the brown adipose tissue (BAT) (Figure 5f). Also, positive staining on a small fraction of individual cells in the lymph nodes and the parotid gland as well as a slight positive cytoplasmic staining for the majority of ducts in the submandibular gland was detected.

Apart from the qualitative detection of transduced cells in tissue samples, quantitative assessment of cell numbers or gene expression levels is important to compare, for example, different therapeutic treatments. Software for automated signal quantification enables a quick and unbiased evaluation of IHC results [21]. However, a high signal-to-noise ratio and staining specificity are prerequisites for reliable results.

**Figure 6:**
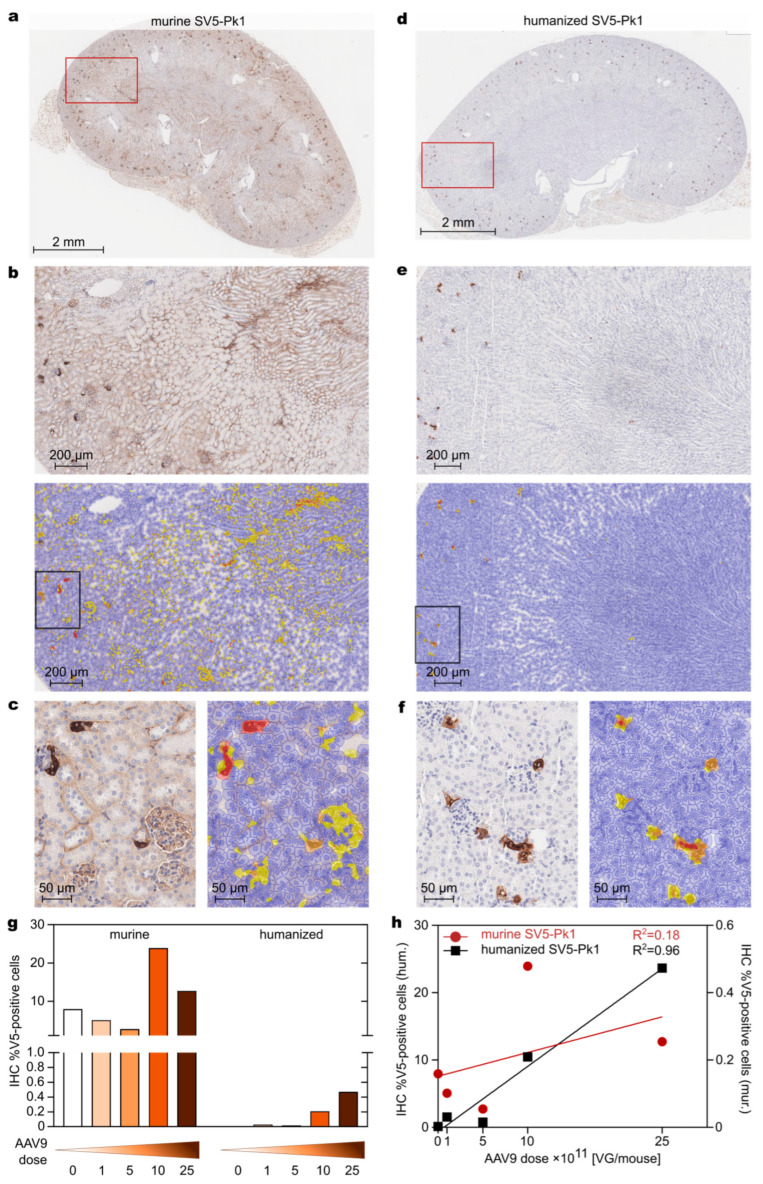
Quantification of AAV9^DTPA-R^ transduced cells in kidneys of C57BL/6 mice. (**a**–**f**) Kidney section of mouse #755 (highest titre, 2.5 × 10^12^ vg/mouse) stained with (**a**–**c**) mu_SV5-Pk1 and rabbit anti-mouse bridging antibody or (**d**–**f**) the hu_SV5-Pk1 and rabbit anti-human bridging antibody. (**a**,**d**) Whole kidney section and (**b**,**c**,**e**,**f**) magnification and corresponding quantification of percent positive cells using QuPath with low (yellow, threshold = 0.2), medium (orange, threshold = 0.4), and high (red, threshold = 0.6) threshold segmentation. (**g**) Quantification of the percentage of V5 tag positive cells by QuPath using all positive cells (threshold = 0.2) in the whole kidney section for tissues stained with mu_SV5-Pk1 or hu_SV5-Pk1 and (**h**) correlation with the AAV9^DTPA-R^ dose applied.

We aimed to evaluate the transduction of kidney cells in C57BL/6 mice injected intravenously with different doses of the AAV9^DTPA-R^ viral vector. As described above, staining with the mu_SV5-Pk1 antibody resulted in a high background signal due to endogenous murine immunoglobulin in blood and on plasma cells visible in the form of large-scale extra- and also intracellular stains (Figure 6a,b and Figure S2). Quantification of positive cell populations in these IHC images using QuPath [21] resulted in false positive results in all parts of the kidney, in particular in the glomeruli and interstitial blood capillaries (Figure 6b,c). Nevertheless, highly positive cells in the medulla could be detected if applying a high threshold for segmentation (Figure 6c), whereas the complete exclusion of non-specific staining in plasma cells was not possible.

In contrast, staining with the hu_SV5-Pk1 antibody led to drastically decreased background staining in the kidney and allowed the exact quantification of cells positive for the V5 tag (Figure 6d–g). Transduced cells were predominantly found in the interstitial compartment surrounding proximal tubules adjacent to the glomeruli, while only a few V5 tag positive cells were detected within the medulla of the kidney (Figure 6d–f). This was most likely due to filtration of the intravenously injected AAV9 particles in the glomeruli, leading to transduction of glomerular cells but not in the downstream tubular cells of the nephron [29].

Quantification of the positive signals in the whole kidney using the murine antibody resulted in a range of 2.7 to 23.9% (false-)positive cells (Figure 6g), whereas the humanized antibody detected only 0.002 to 0.4% positive cells (Figure 6g), thus substantially reducing false-positive signals. The impact of the much lower background also became apparent when correlating the percentage of positive cells with the dose of AAV9^DTPA-R^ injected. The false positive results from the tissues stained with the murine antibody led to a random distribution of values without a meaningful correlation with the applied dose (R^2^ = 0.18) (Figure 6h). In contrast, the IHC quantification results obtained with the hu_SV5-Pk1 antibody led to a good correlation with the AAV9 dose (R^2^ = 0.96) (Figure 6h).

### 3.4. Human Tissue Staining

**Figure 7:**
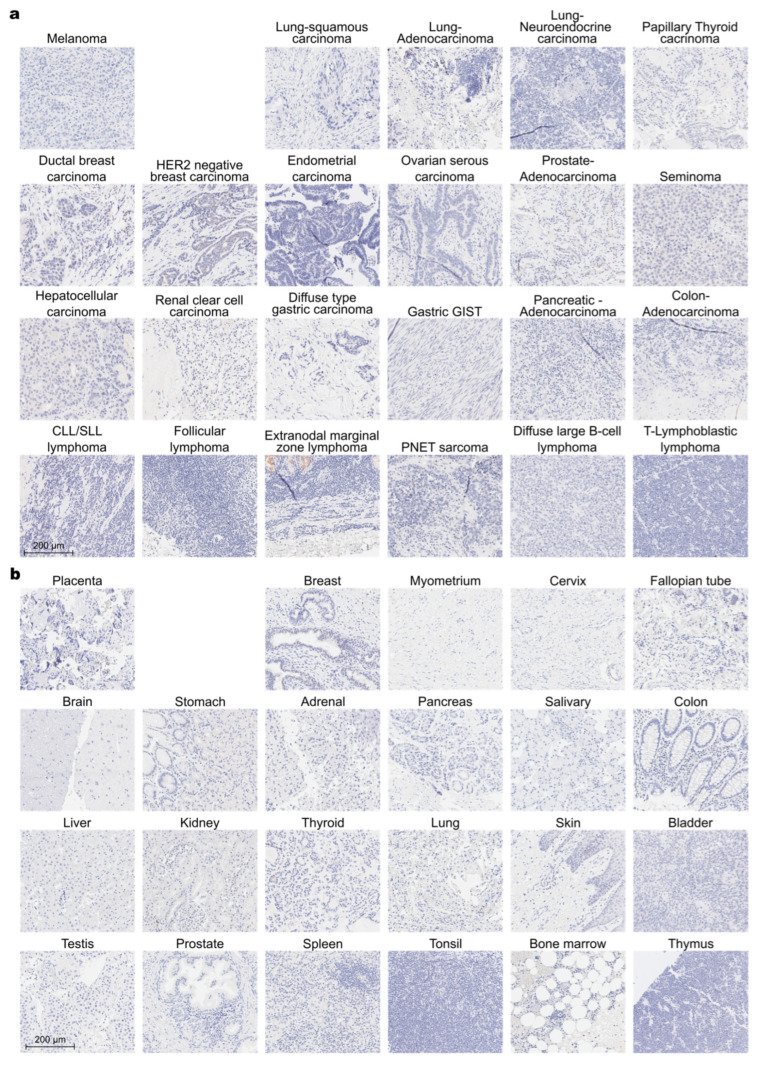
Specificity evaluation of the anti-V5 tag antibody on tissue microarrays. Commercially available tissue microarrays with (**a**) human cancer tissue and (**b**) human normal tissue were stained with the mu_SV5-Pk1 and rabbit anti-mouse bridging antibody, showing no specific staining of SV5-Pk1, indicating the absence of unspecific binding by the antibody.

Finally, to further evaluate the potential cross-reactivity of the murine and humanized versions of the SV5-Pk1 antibody with unknown endogenous epitopes, a wide range of human tissues, both normal and cancer tissues, were analyzed using commercially available tissue microarrays (TMA). As seen before, when using an antibody originating from the same species as the tissue stained, here hu_SV5-Pk1, endogenous immunoglobulin led to a high background signal (Figure S3a,b). As expected, this effect was not observed when using human cancer cell lines cultured in vitro, which did not reveal relevant unspecific binding of the hu_SV5-Pk1 (Figure S4). The mu_SV5-Pk1 antibody, on the other hand, did not show binding on any of the human TMAs, thus highlighting the pronounced epitope specificity of the anti-V5 antibody in a wide array of tissues (Figure 7a,b, and Figure S5).

## 4. Discussion

Epitope tags are well-established tools enabling the reliable detection and also purification of fusion proteins [3]. Compared to tag-free proteins, generating individual antibodies or establishing protocols for each target is unnecessary, which simplifies research [30]. Nevertheless, the impact of incorporating an epitope tag into a biologically active protein or receptor has to be carefully considered. Previous studies indicated that the insertion of an epitope tag can reduce the protein expression levels. For example, the Flag-tag and the myc-tag reduced the expression of neuronal nicotinic receptors, whereas the V5 tag had only a minor impact [31], possibly due to its lower number of charges at physiological pH (see Table S1). Furthermore, the hydrophobicity or hydrophilicity of an attached epitope tag, indicated by the GRAVY value, may impact protein solubility, folding or transport [2]. In fact, strongly lipophilic sequences tend to integrate into the membrane [2] or to form aggregates [32,33]. Different epitope tags have been explored to purify and study CAR-T cells, including the His_6_-tag [34], myc-tag [35,36,37], Flag-tag [38,39] and the *Strep*-tag II [40,41]. The low number of charges at physiological pH, the predominantly hydrophilic character of the V5 tag, and the availability of high-affinity antibodies make this tag promising for studies of cell membrane-bound proteins, such as the reporter protein DTPA-R [19].

However, cell or tissue processing, like enzymatic digest or fixation, may affect the epitope tag and limit its utility. In particular, extracellularly exposed proteins might be enzymatically cleaved upon cell detachment or masked by the fixation chemistry, thus resulting in lowered signals or even false negative results. As demonstrated in this study, the V5 tag proved to be resistant to trypsin, as expected from its sequence, which lacks arginine residues and exhibits only one lysine residue preceding the imino acid proline, thus constituting a poor substrate for this protease. On the other hand, the V5 tag is susceptible to cleavage by other common enzymes. For comparison, the Flag-tag, *Strep*-tag II, myc-tag, AU1-tag, E-tag, and ALFA-tag all contain cleavable lysine or arginine residues (see Table S1), thus rendering trypsin, the standard protease for cell detachment, problematic for sample preparation. Apart from that, we observed that chemical fixation reduces the recognition of the V5 tag by the mu_SV5Pk1 antibody; however, this effect is well-known for different fixatives and also for other tag systems. Therefore, impaired detection efficiency must be considered in general if cell fixation is required [42,43,44].

IHC is a standard method relying on the sensitive detection of an epitope within tissue slices in the presence of a low background signal. Due to the availability of high-affinity antibodies, the V5 tag offers promising characteristics in this regard. When searching for the central sequence motif of the V5 tag, IPNPLLGL, which is tightly bound by the mu_SV5Pk1 antibody [9], in the human or murine proteome databases using BLASTP [45], no relevant hits were found. Together, this should warrant a good signal-to-noise ratio, which is especially important for the detection of cell populations with low expression of the tag in a tissue. Nevertheless, we observed unspecific background staining with the mu_SV5-Pk1 antibody in liver and kidney tissue from CD1-nude mice transduced with AAV9^DTPA-R^ viral particles, complicating analysis and hindering automated quantification. This might be due to the binding of the murine antibody by murine Fc receptors [46] or due to the presence of endogenous murine IgG and IgM antibodies within the tissue sample, which were recognized by the secondary staining reagent used in conjunction with the murine primary antibody [27,47]. A major influence from Fc-gamma-receptor (FcγR)-mediated binding seemed unlikely, as FcγRs are expressed on the cell surface. At the same time, the observed background staining was mostly acellular (see Figure 5a,b and Figure 6a,b). Furthermore, even though human Fc-gamma-receptors also bind murine IgG2a antibodies (like mu_SV5-Pk1) [48], we did not observe background staining when using the mu_SV5-Pk1 antibody on human tissues, even though tissues like spleen or tonsils contain FcγR-expressing cells [49,50]. Also, murine FcγRs cross-react with human antibodies [51], still, we observed no background staining using the humanized anti-V5 tag antibody on murine tissue. Summarizing these observations, we conclude that the observed background staining is most likely due to endogenous IgGs and IgMs in the sample tissues detected by the secondary reagents. Similar observations were reported by others for the IHC of murine tissues using murine antibodies [52].

To overcome this limitation, we employed the recently described humanized SV5-Pk1 antibody [28], thus permitting the use of secondary reagents that do not match the species of the research animal. Using the hu_SV5-Pk1 antibody, exhibiting a low K_D_ value of 571 pM [28], led to a pronounced reduction of background signal on mouse tissue and allowed the precise quantification and localization of V5 tag-positive cells. This considerable improvement in IHC detection became especially evident when analyzing the axial cross-section of a mouse head after transduction with AAV9^DTPA-R^ viral vectors, which showed large areas with false-positive signals when using the mu_SV5-Pk1 antibody. In contrast, the unambiguous identification of transduced cells exhibiting the V5 tag was only possible in the tissue slice if stained with the hu_SV5-Pk1. Using this antibody, we observed transduction of individual adipocytes in the BAT in the neck region upon intravenous injection of AAV^DTPA-R^, in line with previous findings [53,54]. Positive staining of individual cells in the lymph nodes may indicate transduction of immune cells accumulating in these tissues [55]. Still, this positive cellular staining could potentially also be due to FcγR-mediated binding of the hu_SV5-Pk1 antibody. While the transduction of acinar cells in salivary glands by AAV9 was reported before [55], this tissue is not a common target of AAV9 [56]. Taken together, the IHC indicated that the BAT is the major contributor to the PET signal obtained. Nevertheless, as different slices in PET showed dispersed signals, and adjacent tissues such as the lymph nodes also showed positive V5 tag staining in IHC, it seems that multiple tissues had contributed to the measured PET signal. Notably, the brain of mice in this study did not show any transduced cells, which is most likely due to the low dose of 4 × 10^12^ vg/kg, whereas a much higher dose of 1 × 10^14^ vg/kg was reported to be necessary for successful brain transduction [57].

To evaluate the specificity of the V5 tag within complex biological samples, human tissues from several normal or cancer types were tested, showing no binding by the murine SV5-Pk1 antibody. These data confirm the high epitope specificity of the V5 tag antibody, suggesting that the sensitive detection and application of V5-tagged reporter proteins should be feasible in a clinical setting. Even though SV5 (also known as parainfluenza virus 5, PIV5) can infect various hosts, including humans, such infections are mostly asymptomatic [58], and the prevalence of neutralizing antibodies against PIV5 in human sera is estimated to be below 30% [59,60]. Moreover, these antibodies mostly target the major surface glycoprotein or the nucleoprotein [61], whereas the V5 tag is derived from the RNA polymerase α subunit [8]. On the other hand, the prediction of immunogenicity using Immune Epitope Database & Tools (IEDB) [62] did not show positive results for the V5 tag in the context of HLA complexes [19], indicating that the V5 tag is unlikely to cause an immune reaction in humans.

## 5. Conclusions

With more recent developments in the field of synthetic biology, such as the construction of CARs, the benefits of the V5 tag system appear attractive for this new class of cell therapies. Together with antibodies having high affinity and specificity, this versatile tag can serve as a surface marker for cell isolation and simplify the detection of cell and gene therapy products ex vivo, thus supporting preclinical research and clinical trials [63]. Currently, CAR-T cell therapy is often monitored in a clinical setting using either flow cytometry or quantitative PCR of blood samples rather than IHC [64]. The latter may become more relevant in the future, for example, when treating solid tumors. There, it can provide spatial information on the presence of transduced immune cells together with the conventional histopathological analysis of the malignant tissue, whereas blood samples can only provide limited information. Detecting CAR-T cells within their spatial context would also enable the exploration of their interactions with various cell types using the latest spatial ~omics methods. As shown in this study, the V5-epitope tag can function as a versatile tool to sensitively detect engineered proteins in preclinical and clinical research, providing a meaningful addition to current methods. Furthermore, the use of the V5 tag would nicely complement our recently described DTPA-R reporter system for the PET imaging of genetically labeled cells [19].

## Data Availability

The original contributions presented in this study are included in the article/Supplementary Materials. Further inquiries can be directed to the corresponding author.

## Supplementary Materials

The following supporting information can be downloaded at: https://www.mdpi.com/article/10.3390/biology14070890/s1, Figure S1: V5 tag IHC of liver from NSG mice with CAR-T^DTPA-R^ cells; Figure S2: V5 tag IHC of kidneys from AAV9^DTPA-R^ transduced mice; Figure S3: IHC of TMAs (human cancer and normal tissue) with hu_SV5-Pk1; Figure S4: IHC of human cancer cell lines with hu_SV5-Pk1; Figure S5: IHC of TMAs (human cancer and normal tissue) with mu_SV5-Pk1; Table S1: Common epitope tags and their biochemical properties.
