# Supplementary material for: The V5-Epitope Tag for Cell Engineering and Its Use in Immunohistochemistry and Quantitative Flow Cytometry
Source: Biology (Basel). 2025 Jul 20;14(7):890. doi: 10.3390/biology14070890 (PMC12292070; doi:10.3390/biology14070890)
Supplement: Supplementary file 1 [file biology-14-00890-s001.zip › biology-3647531-supplementary.pdf]

The V5-Epitope Tag for Cell Engineering and Its Use in Immunohistochemistry and Quantitative Flow Cytometry

Supplementary materials

Table S1. Common epitope tags and their biochemical properties.

**Tag**	**Seqeuence**	**Origin**	**Amino acids**	**Charge at pH 7.4 (-/+)**	**Theo. pI**	**GRAVY**
HA	YPYDVPDYA	Amino acids 98-106 of Human influenza	9	2/0	3.56	-0.900
Myc	EQKLISEEDL	Amino acid residues 410-419 of Human c-Myc	10	4/1	4.00	-1.010
Flag	DYKDDDDK	Synthetic	8	5/2	3.97	-3.325
V5	GKPIPNPLLGLDST	Amino acid residues 95 to 108 of the P-subunit of the viral RNA polymerase	14	1/1	5.84	-0.150
AU1	DTYRYI	Major capsid protein of bovine papillomavirus-1	6	1/1	5.83	-1.133
His_6	HHHHHH	Synthetic	6	0/0	7.21	-3.2
Strep-II	WSHPQFEK	Synthetic	8	1/1	6.75	-1.825
KT3	KPPTPPPEPET	From simian Virus 40 (SV40) large T antigen	11	2/1	4.53	-1.991
E-tag	EKEALKKIIEDQQESLNKW	Synthetic	19	5/4	5.01	-1.416
ALFA	PSRLEEELRRRLTEP	Synthetic	15	4/4	6.72	-1.687

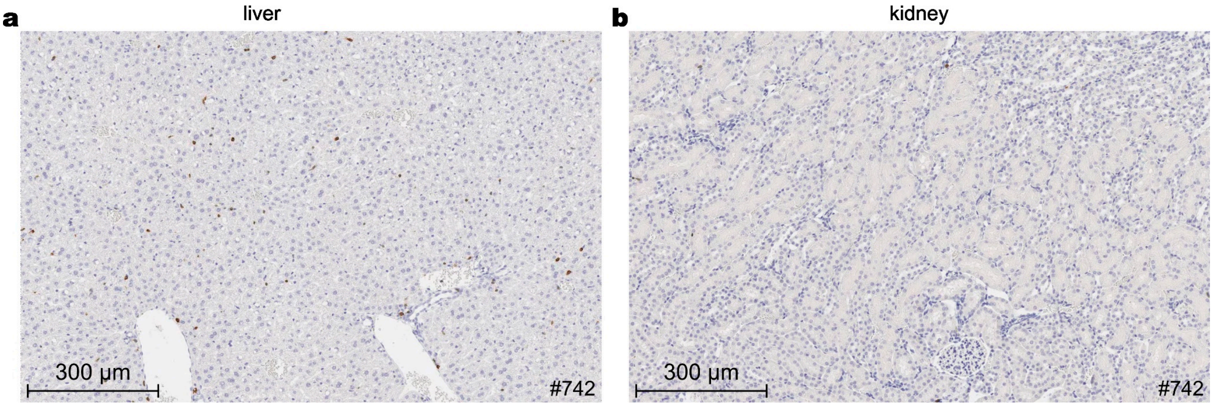

Figure S1. V5 tag IHC of liver from NSG mice with CAR-T^{DTPA-R} cells.

NSG mice bearing a Raji lymphoma were injected with CAR-T^{DTPA-R} cells, sacrificed 31 days p.i. and liver (**a**) and kidney (**b**) were fixed in formalin for IHC analysis. Tissue slices were stained with mu_SV5-Pk1 antibody and secondary anti-mouse antibody showing few V5 tag positive CAR-T cells but no background signal.

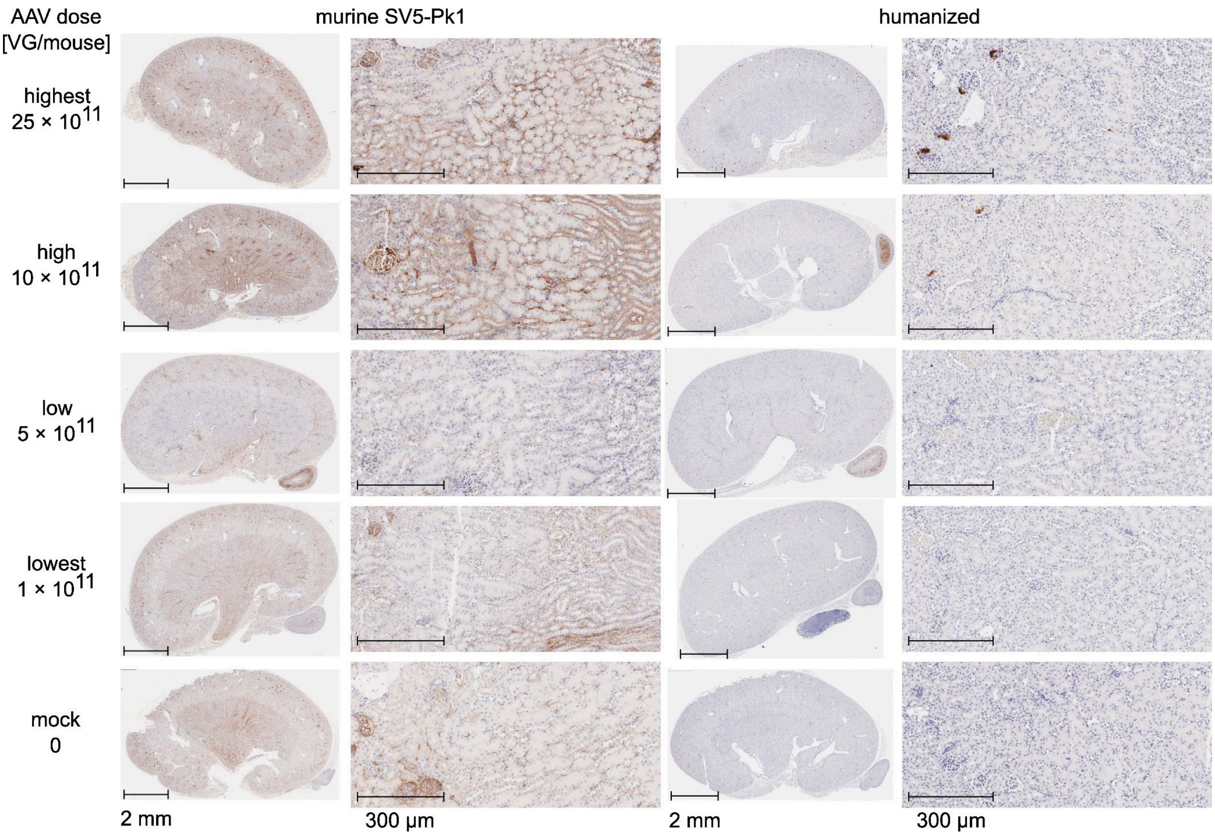

Figure S2. V5 tag IHC of kidneys from AAV9^{DTPA-R} transduced mice.

C57BL6 mice were i.v. injected with AAV9^{DTPA-R} viral vectors (doses 1 to 25 × 10^{11} vg/mouse) and sacrificed 7 days p.i.. Unspecific background staining on formalin-fixed kidneys with the mu_SV5-Pk1 (left) was not detectable when using the hu_SV5-Pk1 (right; DAB, brown).

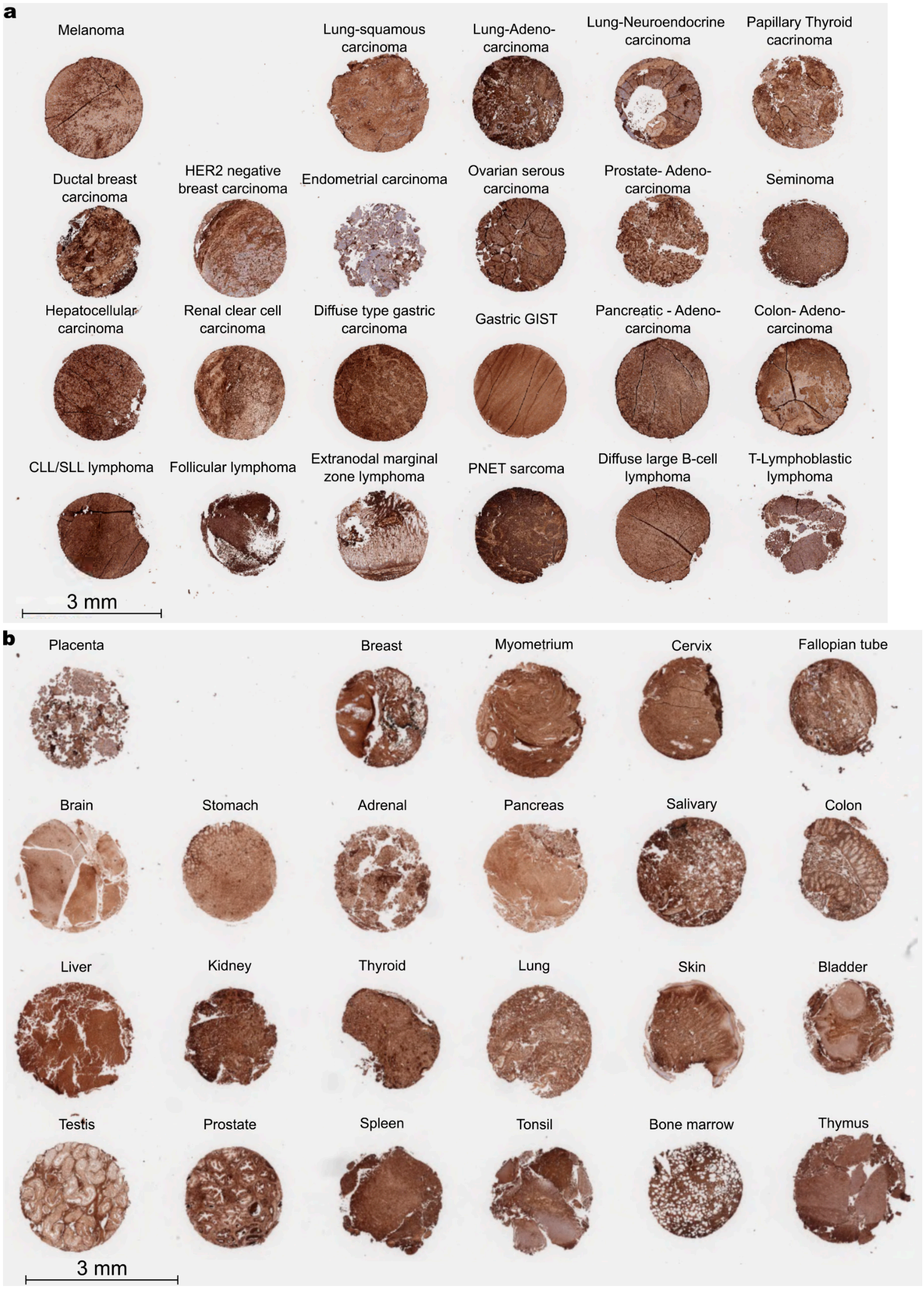

Figure S3. IHC of TMAs (human cancer and normal tissue) with hu_SV5-Pk1.

TMAs of human cancer tissue (**a**) and human normal tissue (**b**) were stained with hu_SV5-Pk1 and rabbit anti-human bridging antibody (DAB, brown) showing high background staining.

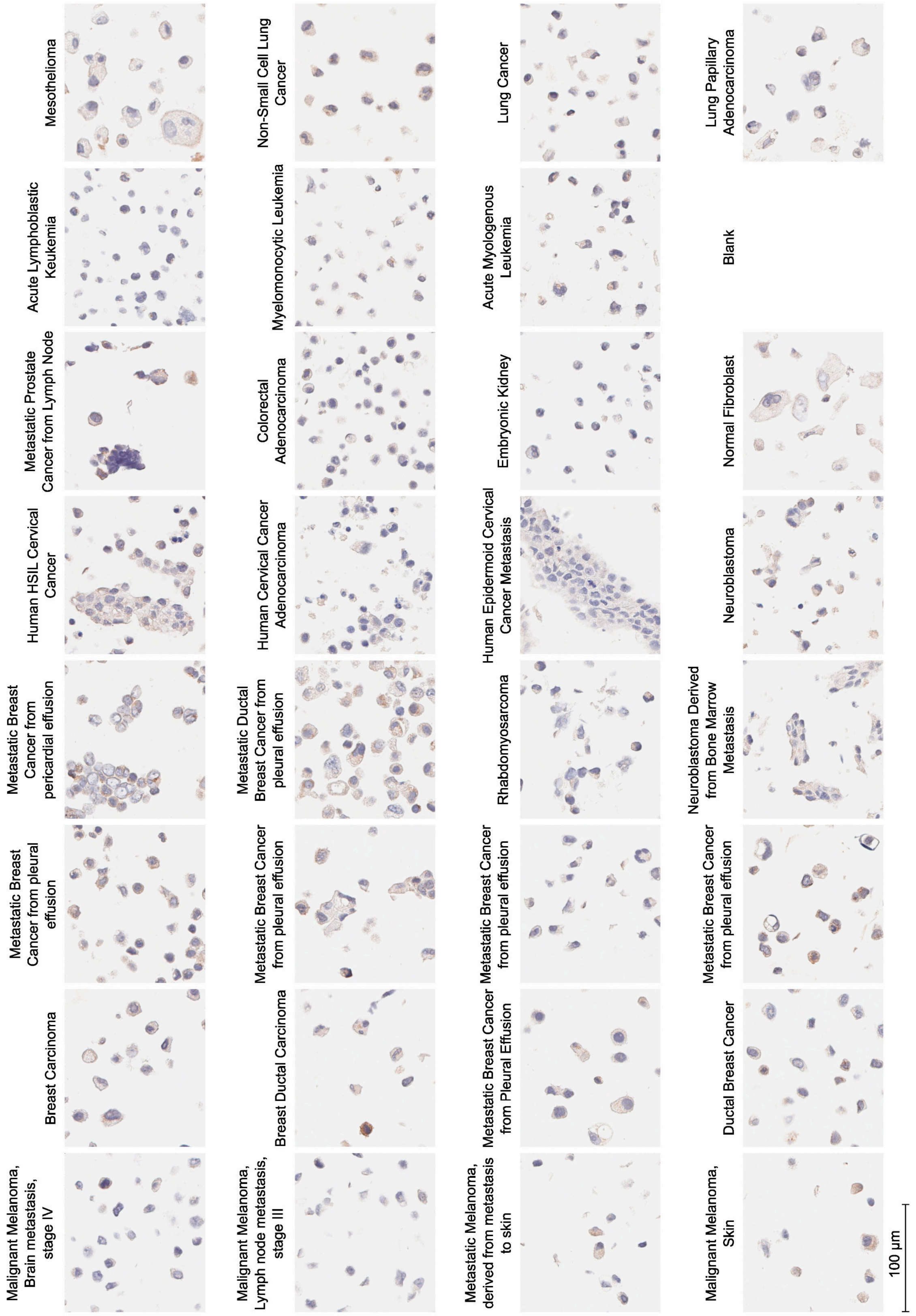

Figure S4. IHC of human cancer cell lines with hu_SV5-Pk1.

Microarray with human cancer cell lines was stained with hu_SV5-Pk1 and rabbit anti-human bridging antibody (DAB, brown) showing no or only very scant signal.

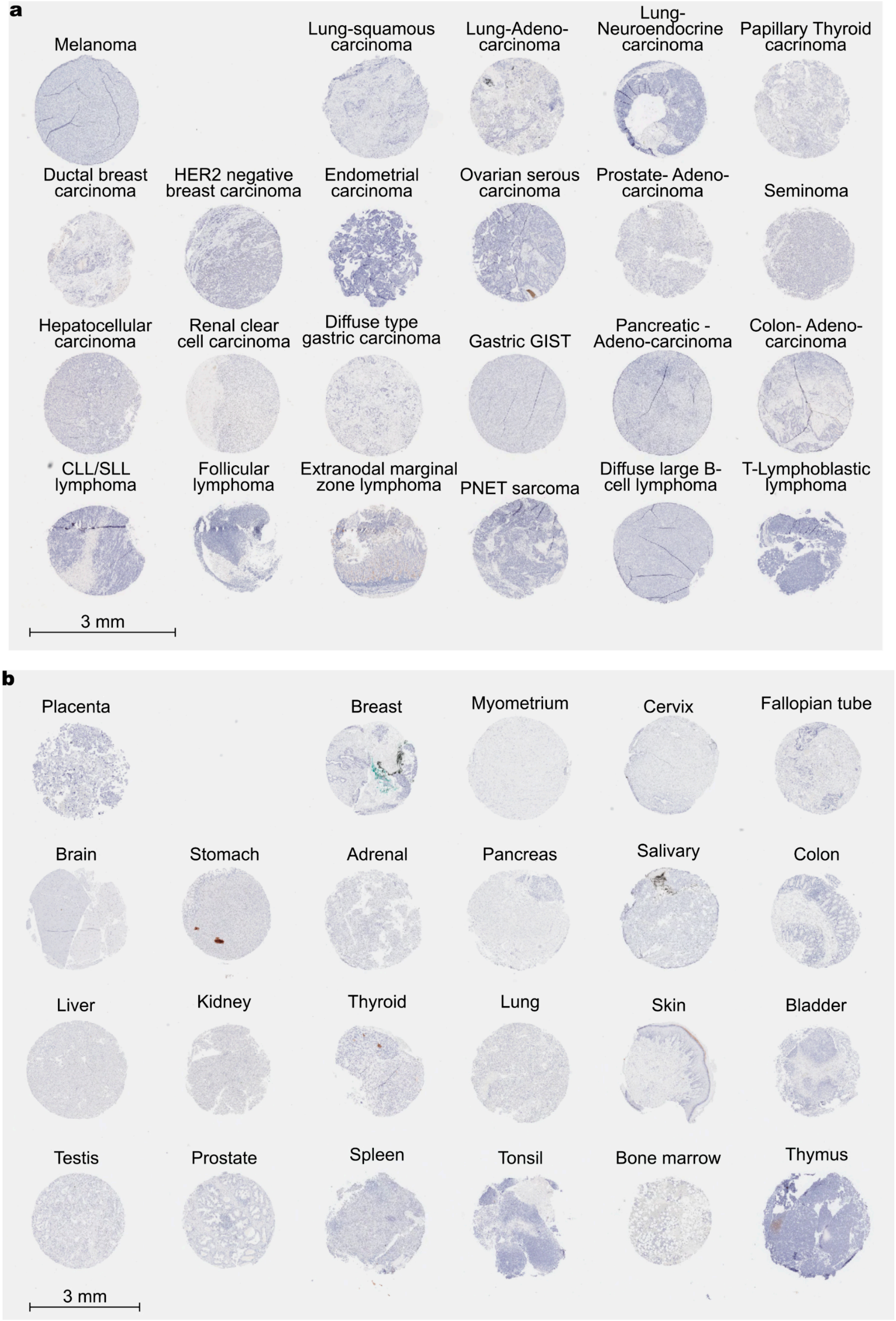

Figure S5. IHC of TMAs (human cancer and normal tissue) with mu_SV5-Pk1. Overview of TMAs of human cancer tissue (**a**) and human normal tissue (**b**) stained with mu_SV5-Pk1 and rabbit anti-mouse bridging antibody (DAB, brown) (see also **Fig. 7**). No specific positive staining was observed.
